# Drs. Keller & Fox

**Published:** 1877-07-20

**Authors:** 


DRS. KELLER & FOX.—See the card of Drs. Keller & Fox, Hot Springs, Arkansas. They are highly intelligent and worthy members of the profession.

